# Transcatheter aortic valve implantation (TAVI) prostheses *in vitro* - biofilm formation and antibiotic effects

**DOI:** 10.1016/j.bioflm.2024.100236

**Published:** 2024-11-01

**Authors:** Torgny Sunnerhagen, Thomas Bjarnsholt, Klaus Qvortrup, Henning Bundgaard, Claus Moser

**Affiliations:** a Department of Clinical Microbiology, Rigshospitalet Copenhagen University Hospital, Copenhagen, Denmark; b Division for Infection Medicine, Department for Clinical Sciences Lund, Faculty of Medicine, Lund University, Lund, Sweden; c Clinical Microbiology and Infection Control, Office for Medical Services, Region Skåne, Lund, Sweden; d Costerton Biofilm Center, Department of Immunology and Microbiology, Faculty of Health and Medical Sciences, University of Copenhagen, Copenhagen, Denmark; e Department of Biomedical Sciences, Core Facility for Integrated Microscopy, University of Copenhagen, Copenhagen, Denmark; f Department of Cardiology, Rigshospitalet Copenhagen University Hospital, Copenhagen, Denmark; g Department of Clinical Medicine, Faculty of Health and Medical Sciences, University of Copenhagen, Copenhagen, Denmark

## Abstract

**Background:**

Transcatheter aortic valve implantation (TAVI) is a percutaneous catheter-based treatment of aortic stenosis as an alternative to open heart valve surgery. In cases of TAVI endocarditis, the treatment possibilities may be limited as surgical removal of the infected valve may be associated with a high risk in elderly, comorbid or frail patients. The propensity of bacteria to form a biofilm on foreign material is assumed to be of importance part of the disease process in TAVI endocarditis, but no studies on biofilm formation on TAVI valves have been conducted. We hypothesize that *Staphylococcus aureus* and *Enterococcus faecalis* biofilm formation on TAVI valves may have an impact on antibiotic tolerance and non-surgical cure rates.

**Methods:**

TAVI valves (pieces including part of the metal frame, approximately 1 cm wide) were exposed to either species *in vitro* in LB-Krebs Ringer medium at 37 °C, with the bacterial count being assessed by culturing of sonicated TAVI pieces and broth at 0, 4, 18 and 24 h after bacterial exposure. Scanning electron microscopy (SEM) was performed. Effects of ampicillin, gentamicin, moxifloxacin, rifampicin (for *S. aureus*), and ceftriaxone (for *E. faecalis*) at 5 times minimal inhibitory concentration were tested alone and in combination with ampicillin. Antibiotics were added to biofilm aged 0 or 24 h and the effects assessed.

**Results:**

Exposure for 15 min established attachment to all of valve pieces. SEM findings were consistent with biofilm formation and suggested lower amounts of bacteria on the metal compared to the tissue part of the TAVI valves. The number of bacteria attached to the TAVI valves increased until 24 h of incubation from less than 10^1 to a level of approximately 10^9 CFU/g. The bacteria became more tolerant to antibiotics on the TAVI valves over time, with the bactericidal effect against 24-h old biofilm being significantly less effective than against 0-h old biofilm depending on antibiotic.

**Conclusions:**

The results indicate that bacteria can adhere to metal and tissue parts of the TAVI valves within minutes after an exposure which is comparable to transient bacteremia *in vivo*, and that the bacteria rapidly gain biofilm properties, associated with significantly reduced antibiotic effect.

## 1 Introduction

The introduction of transcatheter aortic valve replacement (TAVI) has made aortic valve replacement possible in a larger proportion of patients with aortic stenosis [1,2]. Though people with contraindications for open surgery remain one of the groups where TAVI is used, the indications have broadened, and younger patients are also undergoing TAVI procedures [3,4]. This means that the total population living with TAVI valves has increased, as has the duration that patients live with their TAVI valves [[5], [6], [7], [8], [9], [10], [11]]. TAVI endocarditis (TAVI IE) is a feared complication of TAVI valve implantation, with studies reporting a 90-day mortality of approximately 25 %, and a 1-year mortality around 40 % studies [[12], [13], [14], [15]]; a Canadian study did however report an in-hospital-mortality approaching 50 % for *Staphylococcus aureus* TAVI endocarditis [16].

*S. aureus* and *Enterococcus faecalis* are among the most common bacteria causing TAVI IE [12,13,17]. These bacteria often form biofilms on foreign material, contributing to the pathogenesis in endocarditis [[18], [19], [20], [21]]. Biofilms can form without the presence of a foreign body such as an implanted prosthesis, such as the proposed process of endocarditis vegetations formed on damaged valves where the biofilm interacts with the thrombus and deposited platelets [18,20,22,23]. Foreign bodies are however generally thought to increase the risk of biofilms formation. Biofilm formation on surfaces is characterized by bacterial adhesion to the surface and to each other, and with a reduced tolerance to antibiotics that is distinct from traditional antibiotic resistance [[24], [25], [26]]. The effects on foreign bodies on the risk of bacterial adhesion, and the risk of endocarditis, is considered by guidelines for both diagnosing and treating endocarditis and in the diagnostic criteria [[27], [28], [29]]. Biofilm formation on the TAVI valves is assumed to form, but models of this have not been explored. Commonly used and recommended antibiotic treatments often combine a betalactam (usually a penicillin) with, fluoroquinolons, rifampicin (for *S. aureus*), aminoglycosides (for *E. faecalis*) or combining an aminopenicllin with ceftriaxone (for *E. faecalis*). Though the combination of a betalactam with an aminoglycoside is not commonly used for *S. aureus*, aminoglycosides are sometimes used in the initial treatment of bacteremia in some settings [[29], [30], [31], [32], [33], [34], [35], [36], [37]].

The purpose of this study was to investigate if biofilm indeed forms on TAVI valve material, and how this would affect the effect of commonly used antibiotics for treatment of TAVI endocarditis, especially the effect of combining antibiotic drugs. As they are among the most common bacteria in TAVI endocarditis *S. aureus* and *E. faecalis* were used in the current model.

## 2 Methods

### 2.1 Assay medium, bacteria, and antibiotics

An assay medium consisting of 50 % Lysogeny broth (SSC Panum, Copenhagen, Denmark) and 50 % Krebs-Ringer buffer (SSC Panum, Copenhagen, Denmark, supplemented with 0.2 % [5.2 mM] d-Glucose Merck, Darmstadt, GER) was used for culturing of the bacteria.

The strains *Staphylococcus aureus* NCTC 8325–4 (a wild type strain susceptible to penicillin) and *Enterococcus faecalis* (sourced from the POET trial [32]) were used, both without acquired antibiotic resistance for the antibiotics used. The minimal inhibitory concentrations (MICs) for ampicillin, moxifloxacin, rifampicin, gentamicin, and (for *E. faecalis*) ceftriaxone were determined with Etests (Biomérieux, Ballerup, Denmark). The bacteria were incubated overnight, shaking 85 rounds/minute at 37 °C, and subsequently diluted in the assay medium as described in previous studies [18,20,38]. These settings were used for all incubations.

Ampicillin (Stada Nordic, Herlev, Denmark), moxifloxacin (Krka, d. d., Novo Mesto, Slovenia), gentamicin (Panpharma GmbH, Trittau, Germany), rifampicin (Sanofi S.A, Paris, France), and ceftriaxone (Fresenius Kabi, Uppsala, Sweden) were diluted in assay medium to a concentration of five times the MIC of the respective bacterial strain (as measured using gradient tests on Mueller Hinton agar, see Table 1), the concentration chosen to ensure eradication of planktonic growing bacteria but also to obtain anti-biofilm effect. These concentrations are similar to peak concentrations obtained during therapy [[33], [39], [40], [41], [42], [43]].

**Table 1:** Antibiotics used and associated MIC values (determined by gradient tests), as well as antibiotic combinations tested. MH = Mueller Hinton agar, MH-F = Mueller Hinton agar with 20 mg/L β-NAD and 5 % mechanically defibrinated horse blood.

	*S. aureus*				*E. faecalis*		
MIC values (mg/L)		Antibiotic	Antibiotic combinations	MIC values (mg/L)		Antibiotic	Antibiotic combinations
MH	MH-F			MH	MH-F		
0.064	0.064	Ampicillin		0.5	1	Ampicillin	
0.125	0.125	Gentamicin	Ampicillin + gentamicin	4	2	Gentamicin	Ampicillin + gentamicin
0.125	0.064	Moxifloxacin	Ampicillin + moxifloxacin	0.125	0.125	Moxifloxacin	Ampicillin + moxifloxacin
0.016	0.008	Rifampicin	Ampicillin + rifampicin	4	1	Ceftriaxone	Ampicillin + ceftriaxone

### 2.2 TAVI biofilm model

For the biofilm model, expired TAVI valves (ACURATE neo2, Symetis, Boston Scientific, Marlbourough, USA) were cut into pieces lengthwise (approximately 1 cm wide). The TAVI valve consists of decellularized bovine pericardium fastened to a nitinol mesh with sutures. The pieces were cut using surgical wire cutters (DP512R B. Braun Medical AB, Danderyd, Sweden), and the procedure was performed in a biological safety cabinet to reduce the risk of contamination. The weight of the pieces was subsequently determined so that normalization by weight was possible, to compensate for small differences in size. Finally, the resulting pieces were put into 6-well-plates (TPP AG, Trasadingen, Switzerland) with 7 ml of assay medium in each well.

Growth characteristics on the TAVI pieces were studied by diluting overnight culture in assay medium, and added to the assay medium in the well so that a final concentration of 10^2^ colony forming units (CFU) per mL was achieved [18,20,38]. The 6-wells plates with TAVI pieces in assay medium and added bacteria were the incubated 15 min, shaking 85 rounds/minute, to achieve bacterial adhesion. The pieces were then washed once in saline and moved to new 6-well plates with 7 mL of assay medium and the incubation continued. To determine the number of bacteria attached to the TAVI pieces, the pieces were washed three times in saline to remove non-attached bacterial sediment. The pieces were placed in 50 mL Falcon tubes with 50 mL sterile saline solution, and sonicated Branson 5210 sonicator (Branson Ultrasonics, USA). The tubes with TAVI pieces were then centrifuged to concentrate the dislodged bacteria in the bottom of the tubes (10 min, 3720 G). The 45 topmost mL were then carefully removed using a serological pipette. The remaining 5 mL of solution was then vortexed, diluted in a 10-fold dilution series, and 100 μL plated on blood agar plates. The following day the colonies were counted to determine CFU/mL in the solution, and the values adjusted for the weight of the TAVI pieces to produce CFU/g.

To determine the impact of the time available for initial bacterial attachment the pieces were challenged with 10^2^ CFU/mL of bacteria. After 5, 15, 30, or 60 min, the pieces were removed from the assay medium, washed once in saline, and transferred to new assay medium. To assess the attachment to different parts of the TAVI pieces, this was done both to TAVI pieces consisting of all constituent parts (pericardium, fastening sutures, and metal), and to pieces taken from the ends of the TAVI implants, consisting of metal only.

### 2.3 Antibiotic challenges

Assessing antibiotic effects in the model system was done by adding 10^8^ CFU/mL of bacteria to the TAVI pieces in assay medium. After the 15-min challenge phase, the pieces were washed once in saline and moved to new assay medium. Either directly (0 h) or after 24 h of incubation in the assay medium, the pieces were moved again to new assay medium with antibiotics (5 x MIC, either one antibiotic or two in combination, see Table 1) and incubated in 24 h. The pieces were then washed three times in saline and sonicated, the solution plated on blood agar, and the CFU/g was determined.

### 2.4 Scanning electron microscopy

Scanning electron microscopy (SEM) was performed at the Core Facility for Integrated Microscopy (CFIM) at the Faculty of Health and Medical Sciences, University of Copenhagen. TAVI pieces were challenged with bacteria at a concentration of either 10^8^ CFU/mL (high inoculum) or 10^2^ CFU/mL (low inoculum) as described above and incubated according to the protocol.

The samples were washed three times in saline placed in 2 % glutaraldehyde in 0.05 M sodium phosphate buffer, pH 7.4, for fixation. Following 3 rinses in 0.15 M sodium phosphate buffer (pH 7.4) specimens were post fixed in 1 % OsO4 in 0.12 M sodium cacodylate buffer (pH 7.4) for 2 h. Following a rinse in distilled water, the specimens were dehydrated to 100 % ethanol according to standard procedures and critical point dried (Balzers CPD 030) with CO2. The specimens were subsequently mounted on stubs using double adhesive carbon tape (Ted Pella) as an adhesive and sputter coated with 6 nm Gold–Palladium (Leica ACE 600). Specimens were examined with a FEI Quanta 3D SEM operated at an accelerating voltage of 2 kV.

## 3 Results

### 3.1 Attachment to TAVI pieces

**Fig. 1:**
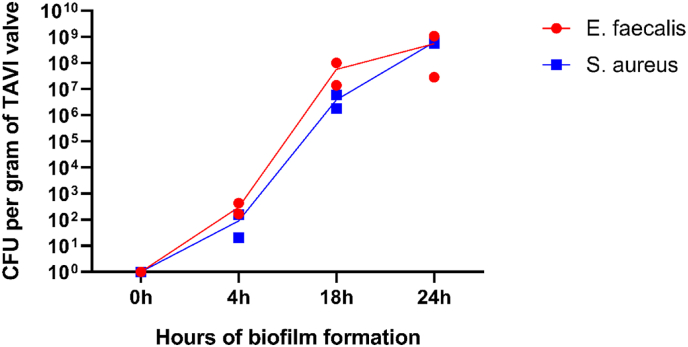
TAVI pieces (n = 2 per timepoint) were exposed to 10^2^ CFU/mL *S. aureus* or *E. faecalis*. After 15 min, the pieces were washed once and moved to new assay medium for 0, 4, 18, or 24 h of incubation. The TAVI pieces were washed three times and sonicated. Bacteria per gram of TAVI piece are shown as detected by quantitative bacteriology. Means and standard deviations are shown. CFU, colony forming unit; TAVI, Transcatheter Aortic Valve Implantation.

Using an inoculum of 10^2^ CFU/ml and a challenge time of 15 min, both *S. aureus* and *E faecalis* were able to reproducibly establish an attachment to the TAVI pieces. The CFU count per gram TAVI valve increased over time to a final level of approximately 10^8^ CFU/g for *S. aureus* and *E. faecalis*. Median CFU/g increased from 10.5 (range <5–435 CFU/g) to 6.4 x 10^7^ (range 1.4 x 10^7^–1.1 x 10^9^) for *S. aureus*, and 63 (range <5–160 CFU/g) to 2.9 x 10^8^ (range 1.8 x 10^6^–6.8 x 10^8^) for *E. faecalis* at 0 and 4 versus 18 and 24 h respectively, p = 0.029 for both bacterial species*.* These bacteria remained attached to the TAVI pieces despite repeated washing in saline (Fig. 1 and Table 2).

Both *S. aureus* and *E. faecalis* attached in all replicates to TAVI pieces containing all components at exposure times of 15 min and more, but not always at exposure times of 5 min. The difference between attachment to metal only compared with pieces containing all components was statistically significant for *S. aureus* at 15 min of exposure, and for *E. faecalis* at 15, 30 and 60 min of exposure, with attachment to the metal being lower (Table 2).

**Table 2:** TAVI pieces, either containing all component or metal part only, were infected with 10^2^ CFU/mL *S. aureus* or *E. faecalis*. After 5, 15, 30, or 60 min of incubation, the TAVI pieces were washed once in saline and moved to fresh assay medium. The pieces were then incubated for 24 h and the presence or absence of bacteria was assessed visually by turbidity.

Exposure time	*S. aureus*			*E. faecalis*		
	Whole TAVI piece	Metal part only	p-value (whole piece vs metal only)	Whole TAVI piece	Metal part only	p-value (whole piece vs metal only)
5 min	1/6	1/6	1	1/6	1/6	1
15 min	6/6	0/6	<0.01	6/6	1/6	0.02
30 min	6/6	4/6	0.45	6/6	1/6	0.02
60 min	6/6	2/6	0.06	6/6	1/6	0.02

### 3.2 Antibiotic tolerance and antibiotic combinations

**Fig. 2:**
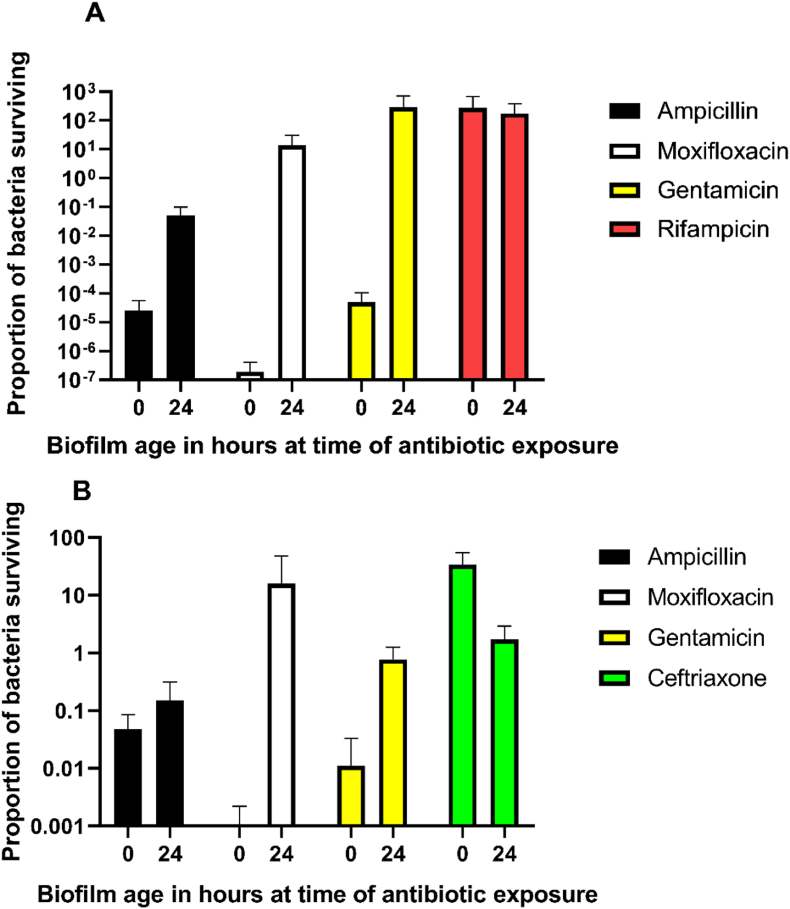
The ratio between the CFU/g for antibiotic treated and the related pretreatment control of TAVI pieces (CFU_after antibiotics at biofilm age X_/CFU_before antibiotics at biofilm age X_). (A) shows values for *S. aureus* (n = 4 replicates). (B) shows values for *E. faecalis* (n = 4 replicates). The X-axis shows the age of the biofilm at the time of starting antibiotic exposure. All experiments used five times the MIC values of the antibiotics tested. 24 h of antibiotic exposure time was used. Means and standard deviations are shown. CFU, colony forming unit; TAVI, Transcatheter Aortic Valve Implantation; MIC, minimal inhibitory concentration.

To evaluate antibiotic tolerance in the model, antibiotics were added after either 0 or 24 h of biofilm formation on the TAVI pieces. A marked increase in tolerance was seen for samples where the biofilm had been allowed to form for 24 h as compared to samples where the antibiotics were added directly after the bacterial challenge (Fig. 2) The exception was when rifampicin was added as the only antibiotic to *S. aureus*. To determine if this was due to the biofilm state as opposed to a direct antibiotic resistance, the bacteria were tested in planktonic phase with gradient tests and antibiotic disks after the exposure. No phenotypically detected resistance was seen, except for rifampicin and *S. aureus*, where there was a consistent development of resistance when rifampicin was used alone.

**Fig. 3:**
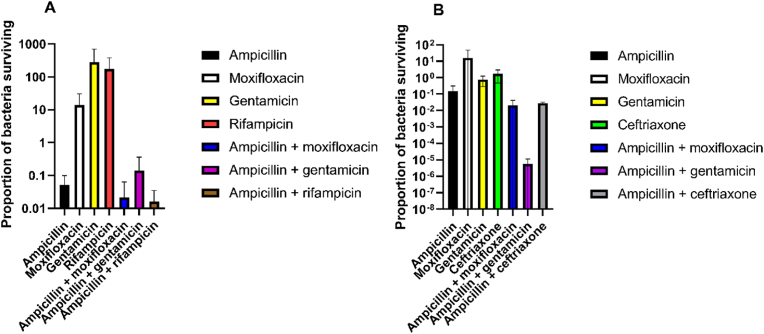
The ratio between the CFU/g for antibiotic treated and the related pretreatment control of TAVI pieces (CFU_after antibiotics at biofilm age X_/CFU_before antibiotics at biofilm age X_). This experiment compared antibiotics in combinations to the single antibiotics, for 24 h old biofilms (A) shows values for *S. aureus* (n = 4 replicates). (B) shows values for *E. faecalis* (n = 4 replicates). All experiments used five times the MIC values of the antibiotics tested. 24 h of antibiotic exposure time was used. Means and standard deviations are shown. CFU, colony forming unit; TAVI, Transcatheter Aortic Valve Implantation; MIC, minimal inhibitory concentration.

Biofilms, grown for 24 h on TAVI pieces were exposed for 24 h to either a combination of ampicillin and either gentamicin, moxifloxacin, rifampicin (for *S. aureus*), or ceftriaxone replacing rifampicin for *E. faecalis*, or to the separate antibiotics alone. There was a tendency towards less bacteria surviving the combination treatment compared to the most effective of the individual antibiotics of the combination, something that was statistically significant for ampicillin combined with gentamicin for *E. faecalis* (p = 0.029), but not for other combinations or for *S. aureus* (Fig. 3).

### 3.3 SEM evaluation of colonized TAVI pieces

**Fig. 4:**
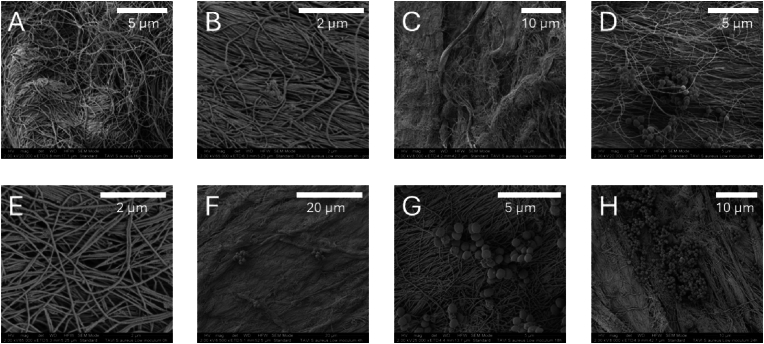
SEM of *S. aureus* with either a high (A–D) or low inoculum (E–H) on TAVI valves (the valve made out of decellularized bovine pericardium being shown). The images show larger amounts and bigger bacterial complexes over time. An inoculum of 10^8^ CFU/mL was used as the high inoculum, and 10^2^ CFU/mL as the low inoculum. (A) and (E) were taken directly after the 15-min exposure of TAVI pieces to the bacterial inoculum, followed by washing the pieces three times in saline. (B) and (F) were recorded after 4 h of incubation, (C) and (G) after 18 h of incubation, and (D) and (H) after 24 h of incubation. Magnifications from 6500 times to 65 000 times. Scale indicated by white bars. CFU, colony forming unit; TAVI, Transcatheter Aortic Valve Implantation; SEM, scanning electron microscopy.

**Fig. 5:**
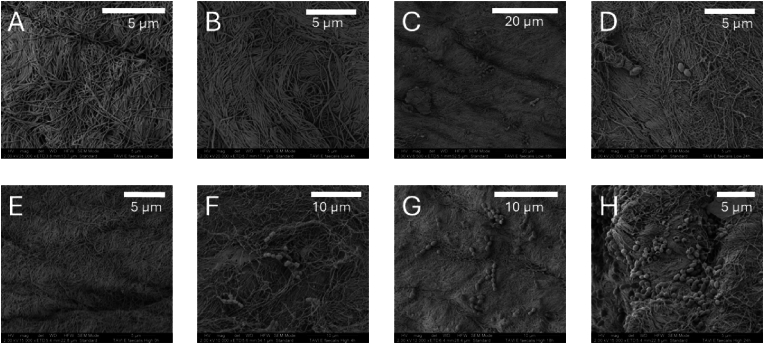
SEM of *E. faecalis* with either a high(A–D) or low inoculum (E–H) on TAVI valves (the valve made out of decellularized bovine pericardium being shown). The images show larger amounts and bigger bacterial complexes over time. An inoculum of 10^8^ CFU/mL was used as the high inoculum, and 10^2^ CFU/mL as the low inoculum. (A) and (E) were taken directly after the 15-min exposure of TAVI pieces to the bacterial inoculum, followed by washing the pieces three times in saline. (B) and (F) were recorded after 4 h of incubation, C) and (G) after 18 h of incubation, and (D) and (H) after 24 h of incubation. Magnifications from 6500 times to 25 000 times. Scale indicated by white bars. CFU, colony forming unit; TAVI, Transcatheter Aortic Valve Implantation; SEM, scanning electron microscopy.

SEM was used to study and confirm bacterial attachment to the TAVI pieces and bacterial organization. The SEM images suggested an increase in bacterial density consistent with the CFU/g as seen by culturing. For *S. aureus,* the higher inoculum of 10^8^ CFU/m induced visible bacterial clusters on TAVI pieces where the bacteria had been allowed to grow for 4 h after exposure, but not at 0 h. For the lower inoculum of 10^2^ CFU/mL, there was visible growth at 18 h of incubation (Fig. 4). The SEM visualization of *E. faecalis* on the collagen matrix of TAVI pieces showed similar results as observed for *S. aureus* (Fig. 5).

**Fig. 6:**
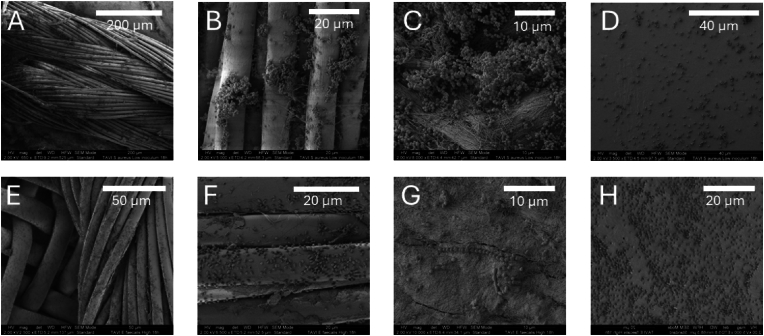
SEM of *S. aureus* (A–D) and *E. faecalis* (E–H) on TAVI. Inoculum of 10^8^ CFU/mL was used, with images recorded after 18 h of incubation. The images show the differences in bacterial attachment and biofilm formation on different surfaces. (A), (B), (E) and (F) display the suture knot fastening the decellularized pericardium to the metal. (C) and (G) display bacteria on the decellularized pericardium. (D) and (H) display the metal. Magnifications from 650 times to 10 000 times. Scale indicated by white bars. CFU, colony forming unit; TAVI, Transcatheter Aortic Valve Implantation; SEM, scanning electron microscopy.

The SEM analysis was suggestive of a greater number of bacteria at the knots fastening the valve tissue to the metal mesh, than to the decellularized pericardium collagen making up the valves and especially compared to the metal. This tendency was more pronounced for *S. aureus* as compared to *E. faecalis* (Fig. 6).

## 4 Discussion

For technical reasons TAVI valves are rarely explanted. Accordingly, *in vitro* studies are essential to improve the understanding of TAVI related endocarditis. Our findings demonstrated that both *S. aureus* and *E. faecalis* can attach to TAVI pieces using concentrations and exposure times that are similar to what are seen during bacteremia. The bacteria remained attached to the pieces when washed and needed sonication to be dislodged, in concordance with biofilm formation. The development of antibiotic tolerance of the bacteria on the TAVI pieces, without acquired antibiotic resistance when the bacteria where exposed to antibiotics in a planktonic phase, also pointed towards biofilm formation. The visualization by SEM showing adhesion and aggregation further strengthens the hypothesis that biofilm formation occurred. Antibiotic tolerance in the biofilm without antibiotic resistance in a planktonic phase is a core concept of biofilm formation, as is adhesion of bacteria to each other and to surfaces, and the formation of aggregates [26].

The fact that both *S. aureus* and *E. faecalis* were able to consistently attach to TAVI pieces consisting of all component parts after 15 min, and sometimes already after 5 min, has implications for the pathogenic process of TAVI endocarditis in the clinic and for further research. The tendency for both species to attach less well to the metal frame when tested using culture, and the SEM seeming to show more aggregates on the knot at the base of the valves was also interesting and seemed to support clinical data regarding the localization of vegetations in TAVI endocarditis [44]. The SEM imaging also showed that the bacteria attached to the surfaces of the TAVI pieces, not only to the edges where the pieces had been cut. As the majority of episodes of endocarditis localized to TAVI valves are generally assumed to be caused by bacteria attaching after a transient bacteremia and rarely in direct relation to insertion, the short exposure needed and the tendency for differential attachment to different parts of the TAVI pieces merit further studies.

Limitations of the study include the use of only two bacterial species, the *in vitro* setting and the use of antibiotics in a way not identical to the pharmacokinetics seen *in vivo*. The model system is also without the platelets and coagulation system that are present *in vivo*. *S. aureus* and *E. faecalis* were chosen as two of the most common species seen in TAVI endocarditis, but the findings in this study cannot be assumed to be representative of other bacterial species. The limitations of the models might not necessarily be identical for *S. aureus* and *E. faecalis*, as the interactions with surfaces, the immune system, and blood components are different for the two species. Examples include staphylocoagulase for *S*. *aureus* and the role of pili in biofilm formation for *E. faecalis* [19,[45], [46], [47]].

The antibiotic effects on the bacteria in LB medium (as was used in this model) might not necessarily be the same as in whole blood, as would be the case in the body. This is also illustrated by the differences between MIC values obtained with gradient tests on agar with and without horse blood added, as the presence of blood components may impact the bacterial behavior. As different manufacturers of TAVI valves use slightly different materials, and different structural solutions, the bacteria-material interaction might also differ. These and other species specific interaction warrant further studies in the context of TAVI endocarditis.

To summarize, in the current model we have shown that bacterial adhesion to TAVI valves happens rapidly in bacterial concentrations similar to what is seen in bacteremia. The model also strongly indicates that biofilm formation occurs *in vivo*. In this experimental model, total bacterial eradication was not seen after 24 h of antibiotic treatment regardless of if the antibiotics were administered in combination or as single treatment. A tendency towards greater bacterial eradication was seen with antibiotic combinations but was in most cases not significant after 24 h. This might be, at least in part, due to the fact that the antibiotic exposure was only for 24 h. In the model used, the time with an ampicillin concentration above MIC was 100 % of the time, leading to a higher bactericidal ampicillin effect than can be expected *in vivo*. This might in turn leading to the addition of another antibiotic to have a lesser effect than expected. The model might be used to test other relevant bacterial species, as well as other antibiotics and non-antibiotic adjunctive treatments. Expanding the model to include blood components to better mimic the *in vivo* situation might also be valuable.

## CRediT authorship contribution statement

**Torgny Sunnerhagen:** Writing – review & editing, Writing – original draft, Visualization, Resources, Methodology, Investigation, Funding acquisition, Formal analysis. **Thomas Bjarnsholt:** Writing – review & editing, Resources, Methodology. **Klaus Qvortrup:** Writing – review & editing, Visualization, Methodology, Investigation. **Henning Bundgaard:** Writing – review & editing, Resources, Methodology. **Claus Moser:** Writing – review & editing, Writing – original draft, Supervision, Project administration, Methodology, Investigation, Funding acquisition, Conceptualization.

## Sources of funding and transparency declaration

10.13039/100016851TS has received funding from the Swedish Society for Medical Research (PD20-0031), Tornspiran Foundation, Royal Physiographic Society of Lund, Swedish Society of Medicine (SLS-971382), Lund University Research Foundations (RMv2021-0002, RMh2021-0001, RMv2022-0004), Thelma Zoégas Foundation for Medical Research (TZ2021-0008), Scandinavian Society for Antimicrobial Chemotherapy (SLS-971424), Längmanska kulturfonden (BA21-0430), Sigurd and Elsa Golje Memorial Foundation (LA2021-0027, LA2022-0068), Mats Kleberg Foundation (2022–109), Direktør Emil C. Hertz og Hustru Inger Hertz' Fond, Arvid Nilssons Fond, the Lars Hierta Memorial Foundation (FO2021-0058), and the Tore Nilson Foundation (2023–126). TB has received honorarium for consultancy from SoftOx and Lohmann and Rauscher. CM has received funding from Novo Nordisk Foundation with ‘Borregaard Clinical Scientist Grant’ (Grant no. NNF17OC0025074), honorarium from GSK and AstraZeneca for scientific presentations, and has received research materials from Boston Scientific International S.A. and for other projects received funding to the department from Reapplix A/S.

## Data Availability

Data will be made available on request.
